# Parenting Science Gang: radical co-creation of research projects led by parents of young children

**DOI:** 10.1186/s40900-020-0181-z

**Published:** 2020-03-02

**Authors:** Sophia Collins, Rebecca Brueton, Tamasin Greenough Graham, Stephanie Organ, Amy Strother, Sarah Elizabeth West, Jean McKendree

**Affiliations:** 1 Lauder, UK; 2 Eastbourne, UK; 3 York, UK; 4 The Science Communication Unit, Faculty of Health and Applied Sciences, Frenchay Campus Coldharbour Lane, University of the West of England, Bristol, BS16 1QY UK; 5 Orpington, UK; 6 Stockholm Environment Institute, Department of Environment and Geography, University of York, York, YO10 5NG UK

**Keywords:** Patient and public engagement, Involvement, PPI, Citizen science, Co-creation, Co-production, Parents of young children

## Abstract

**Background:**

Parents are increasingly searching online for information supported by research but can find it difficult to identify results relevant to their own experiences. More troublingly, a number of studies indicate that parenting information found online often can be misleading or wrong. The goal of the Parenting Science Gang (PSG) project was to use the power of the Internet to help parents ask questions they wanted to have answered by scientific research and to feel confident in assessing research evidence.

**Methods:**

By using Facebook to recruit groups and facilitate interactions, PSG was able to engage fully the target public of parents of young children in the radical co-production of scientific studies, while not creating an undue burden on time or restricting participants due to disability, financial status or location. By giving parents true partnership and control of creation of projects, PSG ensured that the chosen questions were ones that were of most relevance and interest to them.

**Results:**

This paper presents a summary of eight projects, with three in more detail, designed and implemented by PSG Facebook groups in collaboration with experts. Most projects had health related themes, often prompted by dissatisfaction with treatment of parents by health professionals or by feelings of being marginalised by pregnancy and motherhood, as well as by the lack of evidence for their questions and concerns. The PSG approach meant that these frustrations were channelled into actions. All eight of the PSG groups engaged in meaningful interactions with experts and co-produced studies with the groups defining the questions of interest.

**Conclusions:**

This radically user-led design meant that the PSG staff and the collaborating experts had to live with a high degree of uncertainty. Nevertheless, PSG achieved its goal of academically productive, truly co-produced projects, but as important were the positive effects it had on many of the participants, both parents and experts. At the point of writing this paper, PSG projects have led to outputs including at least eight papers published, in press or in preparation, seven conference presentations, testimony to the Infant Feeding All-Party Parliamentary Group, and with more to come.

## Plain English summary

Parents are increasingly searching online for information supported by research but find it difficult to find results relevant to their experiences. The goal of the Parenting Science Gang (PSG) project was to use the Internet to help parents ask the questions they wanted to have answered by science. Using Facebook, PSG engaged parents of young children in the radical co-production of studies, while not creating undue burden on time or restricting participants due to disability, financial status or location. By giving parents control, PSG ensured that the chosen questions were ones that were of most interest to them. This paper presents eight projects designed by PSG Facebook groups in collaboration with experts. Most projects had health related themes, often prompted by dissatisfaction with treatment by health professionals or feelings of being marginalised by motherhood, as well as by the lack of evidence. The PSG approach channelled these frustrations into actions. All eight PSG groups successfully collaborated with experts to co-produce studies for their questions. This radically user-led design meant the PSG staff and the experts had to live with a high degree of uncertainty. Nevertheless, PSG achieved its goal of academically productive projects (with at least eight papers published or in preparation, seven conference presentations and testimony to the Infant Feeding All-Party Parliamentary Group), but as important were the positive effects it had on many of the participants, both parents and experts. For example, parents said their science skills and their confidence had increased.

## Background

### Parenting and the quest for evidence and support

The transition to parenthood can be a stressful and anxious time [1–5] with parents often feeling unprepared for their new roles [6]. Previously, this transition was considered to start from pregnancy, ending a few months post-birth; however, it is now considered to start from the time a couple decides to become pregnant through to the child reaching between the ages of two and three years [6, 7]. Parenthood has been highlighted as one of the most challenging ‘life transitions’ resulting in major changes to the lives of parents [2, 8, 9] and potentially major impacts on parental well-being, parenting quality and infant development [2, 10–14]. Additionally, feelings of isolation and lack of support can be detrimental to the mother, child and wider family [15].

A decrease of ‘in real-life’ support networks (due to factors including the increased mobility of people in our present culture, cuts to services such as children’s centres and other parental support mechanisms) can increase the risk of further isolation and shrinking life-world of parents, mothers in particular [9, 16]. Parenting groups can provide opportunities for peer socialisation, help to diminish feelings of isolation and benefit parental mental well-being [17]. Parents are increasingly turning to more easily accessible virtual communities to seek information and support [6, 18, 19]. This online support can improve parents’ ability to cope with parenting, negate feelings of isolation, increase wellbeing and allow the sharing of experiences, important in developing identity and therefore supporting the transition to parenthood [9, 18, 20–22]. Mothers participating in Internet-based discussion boards found that the online support encouraged them to take more responsibility in parenting and these websites enabled them to recognise their own expertise and knowledge [22]. Social support has been seen to provide information, encourage information-seeking behaviours [23] and has protective effects against negative health consequences and stressful life events, known as ‘stress buffering’. A lack of social support has been shown to be associated with anxiety, depression and have a negative impact upon the immune system [2, 23, 24].

Many women are active seekers of online information, especially in the areas of health, and this increases with the transition to parenthood [18, 19, 25]. Online information-seeking behaviour amongst women during pre- and post-natal stages is high [19, 26] with women twice as likely to look for information online regarding their children compared to men [18]. The Internet as a resource provides immediate, anytime and anywhere support and information, tailored to some extent to the specific needs of the user [27, 28]. This is an important and increasingly common reservoir of informational and social support as parents find more traditional avenues of face-to-face contact with healthcare professionals insufficient in terms of contact time, information provision and emotional support [2, 6, 23, 28–31].

However, it would be erroneous to believe that parents are relying on the Internet exclusively. The Internet is often used to gather new and supplementary material to inform decision-making and improve parents’ ability and confidence in speaking to healthcare professionals and provide reassurance and support to parents between healthcare appointments [23, 28, 32]. This can be mutually beneficial to healthcare services and parents, since mothers desire more support from healthcare professionals but healthcare professionals are increasingly constrained regarding time and other resources and unable to provide the desired levels of contact [2, 6, 28–30, 33, 34]. Disseminating parenting information via the Internet has been considered beneficial by health professionals as it allows supplementary information to be provided, giving parents a better understanding of the choices available to them and aiding informed decision-making about their health care [9, 28, 35]. Other benefits reported for online information and interaction for parents include reaching a wider audience and increasing access to governmental, university and volunteer organisations without increased costs [18].

Nevertheless, while parents are increasingly searching online for experience-based information supported by research or experts, they can find it difficult to find relevant information when searching the plethora of online resources [18, 27, 28, 36]. A perhaps more troubling issue is that a number of studies indicate that parenting information found online can be misleading or wrong, depending on the source [18, 37]. This result is reflected in medical information found online more broadly, where information accessibility and accuracy can vary widely based on source and intent, with media and privately owned sites often being less accurate than academic, charitable or government sources, and higher accuracy sites having worse (i.e. more difficult) readability scores [38–40]. Promoting confidence in finding, interpreting, and critiquing online evidence is vital for helping parents over the hurdles to making informed decisions [41].

On top of the variable accuracy and the difficulty of finding and deciphering online information, parents are often sleep-deprived, time poor and bombarded with advice from family, friends, health care professionals, online forums, popular media, parenting books and other sources that often offer little evidence and can be contradictory. They can feel overwhelmed but at the same time under-informed in areas where they want to have enough evidence to support their decision-making about parenting choices [42].

The goal of the Parenting Science Gang project (PSG), funded by the Wellcome Trust, was to help parents of young children to ask the questions that they want to have answered by scientific research and to feel confident in understanding and assessing research evidence. By giving the parents this opportunity, the project ensured that the chosen question was one that was of relevance and interest to the groups rather than only to the researcher. Following the International Association for Public Participation (IAP2)‘s Public Participation Spectrum, which classifies the public’s role in public participation processes into a spectrum from Inform, through Consult, Involve and Collaborate to Empower, the PSG project took a radical position on public involvement with the intention being that the target public, in this case the parents, would be *Empowered* to define the research questions and the collaborating experts would implement what they decided or would facilitate them to do the study themselves [43]. We also drew on the citizen science literature, and define the PSG project as a Radical Co-created project. Co-created citizen science is where community members and scientists work together through all (or most) stages of the scientific process, which contrasts with contributory citizen science projects where community members only get involved in collecting data and collaborative citizen science projects where community members collect data and engage in one or two other aspects of the scientific process [44]. We call our approach Radical because the project was entirely led by parents, bringing in scientists as ‘experts’ or advisors where needed. The project could also be termed ‘co-production’ of research, as we placed members and ‘experts’ on an equal footing throughout the research project [45]. Parents were also involved in the design of PSG as discussions with members of a precursor project helped inform the design of PSG, and several members helped secure funding for PSG from Wellcome. Throughout PSG, we consulted parents about ongoing project design, for example, format and timings of events. None of the parents was involved in the writing of this paper.

The aim of this paper is to present the methods we used within PSG and some of our findings, in order to inspire others to use similar approaches to engage parents in future research. We describe the radical approaches we used to involve participants, and demonstrate that this approach to conducting research has significant benefits for both project participants and scientific research: our parent groups all identified gaps in existing research which mattered to them, and began to fill them.

## Method

Parenting Science Gang had the aim of changing participants’ relationship to science by involving them as true partners. Parents of young children, the target “public” or “publics” determined what topics and questions they wanted to see addressed and then contributed directly in partnership with identified experts to the research process. Unlike some co-production initiatives that require significant commitment of time by those members of the public who are shaping the research [46], we also wanted every parent to be able to choose how much or how little to contribute, and for parents to be able to participate from their homes as new parents are often time-poor, and struggle to come to real-world events, especially in the evenings. This radical co-creation of research projects in which the target public truly is the lead on the research requires strong commitment and flexibility from the supporting staff and the “experts” who will be directed in their research by the participants.

### Staff in PSG

Of the authors, five were funded staff members for Parenting Science Group (SC, AS, TGG, RB, SW). The grant is held by a social enterprise run by SC. SC has a background in public engagement with science and directed and devised the project. AS has a background in pharmacy and clinical trials and an MBA, and was the project manager. RB and TGG were the project co-ordinators and did most of the communication and management of the groups and liaising with researchers. TGG had a background in science education and outreach, primarily physics. RB had a background in online marketing and community management. SW is a citizen science practitioner and researcher and conducted the project evaluation, along with JM (maternity cover for SW). The five funded members handled all the grant management, coordination and organisation for all the PSG projects, and data collection for evaluation, aside from some in-depth interviews carried out by SO as part of her MSc in science communication.

All authors aside from JM and SO were also members of PSG Facebook groups.

### Recruitment of PSG Facebook groups

To achieve these goals, PSG project leads worked primarily with parenting groups on Facebook, supporting each group of parents to choose their own research question, through a structured process, and then in collaboration with scientists who were approached based on the topic of interest decided by the group, to design and run their own experiment to answer that question. The PSG project took place primarily via Facebook groups. Although targeted at parents of any gender, the vast majority of members were women, most likely because they tend to take on more of the mental labour of parenting [47]. Eight main PSG groups took part over two years with four groups running initially and another four beginning in the second year (see Table 1). In the first year, the groups were spin-off groups created from members of existing Facebook groups we had developed a relationship with through running Nappy Science Gang (a precursor project to PSG, which was a pilot for some of the methods used in this project, see [48]). We termed the existing Facebook groups “mother” groups and our spin-off PSG groups “daughter” groups. In the second year, four more groups were created using a mixture of methods (see Table 1 for details). We also attempted to engage with under-represented groups (including more ethnically diverse parents) by asking PSG members for suggestions and introductions and by approaching relevant organisations, but unfortunately this did not yield any additional groups.

**Table 1:** Parenting Science Gang Facebook Groups in Year 1 and 2, including description of how they were recruited

Facebook Group Name	Year	Origin of Group
Breastfeeding Older Babies and Beyond (BOBAB)	1	SC was a long-standing member of this group, thought it would be a good fit for the project, and approached the admins and had a number of discussions leading to a partnership
UK Breastfeeding and Parenting Support (UKBAPS)	1	Nappy Science Gang active member was an admin and suggested the collaboration
Science-Aware Natural Parenting (SANP)	1	Contact from Nappy Science Gang
Dumfries and Galloway Bumps Babies and Beyond (DGBBB)	1	An active member of Nappy Science Gang was a member of this group and suggested it
Mealtime Hostage (MH)	2	Applied to become a group via a process advertised on our website and Facebook page
Breastfeeding Health Care Experiences (BF HCE)	2	Discussions within PSG BOBAB and UKBAPS groups led to the creation of this group which then recruited members from other PSG groups, and other interested parenting groups
Let Toys Be Toys (LTBT)	2	RB member of LTBT and invited them to collaborate
Big Birthas (BB)	2	SC met at a conference

Two of the year one groups, Breastfeeding Older Babies and Beyond (BOBAB) and UK Breastfeeding and Parenting Support (UKBAPS), had a topic-focussed interest in breastfeeding, while others existed as an online forum for those more broadly interested in a scientific approach to parenting, Science-Aware Natural Parenting (SANP), or for those in a similar locality, Dumfries and Galloway Bumps Babies and Beyond (DGBBB). We found that the groups united by a common interest found it much easier to choose a research question and work together to answer it. We therefore deliberately chose groups with a common interest in the second year. The groups in each year are shown in Table 1, along with how the groups originated.

On joining the daughter Facebook groups, all members were asked to read a statement about the project and take part in a pre-project questionnaire, which collected data about demographics, their science capital, where they gained information from, and what they wanted to gain from the project. Members of the group were regularly reminded to complete the survey if they had not already done so, in order to capture as many of the participants as possible. At the end of the project, members were asked to complete the survey a second time, in order to capture any changes during the course of the project. Other evaluation activities included short questionnaires to experts after online Question and Answer (Q&A) sessions.

### Research co-design and support activities

Most activities took place in these Facebook groups using discussion threads and some polls. Working groups also sometimes met in ‘Private Message threads’ in Messenger. All of our groups began as ‘public’ groups, however, every group independently decided they would prefer the group to be ‘closed’, meaning only members can read the members’ comments and other content within the group.

The main purpose of the PSG project was to put parents in charge of the research agenda, and to identify parenting questions that matter to them and that have not thus far been studied by science, and design and run a research investigation to answer it. The groups broadly followed the approach below, although the influence of personal experiences heavily influenced the chosen research question depending on the group.

1. Questions thread - members contributed questions to which they wanted answers
2. Discussion - questions discussed within the group and in Question and Answer sessions (Q&As) with relevant experts, to identify those which had been answered by research already and to highlight ways of investigating others
3. Top 10 - questions narrowed down to a top ten which could then be discussed in more detail. These ten questions were then featured in the relevant group, one at a time, with prompts to consider the practicalities of answering them experimentally. This was based on our experiences with the precursor Nappy Science Gang project, to avoid groups choosing big, impressive sounding questions which would be experimentally intractable.
4. Range voting - members voted on the top ten questions by range voting (members vote for three choices, giving three votes to their top preference, two to their second, etc). This was chosen to avoid ‘first past the post’ and identify the most satisfactory option for the greatest number of voters.

Once a question had been chosen, members worked together to design an experimental protocol, either in collaboration with a scientist, with advice from a scientific advisor or with ad hoc Q&As for advice on specific areas (e.g. research methodology). Numerous online Q&As were held at scheduled times for an hour, primarily with an invited expert, either to help the group to plan their research or occasionally for general parenting interest. Sometimes this would lead to a research collaboration. Draft protocols were then sent to ‘peer reviewers’ who were other scientists we had identified through Q&As or on the advice of Q&A partners, for feedback. The reviewers’ comments were incorporated by the groups into a final protocol. Depending on the topic chosen, the implementation of the project ranged from the whole project being conducted by the group (BB) to having to substantially hand over to the scientists with support from members (BOBAB/UKBAPS breastmilk study). At all times, the scientists were in discussion with the group members and PSG facilitators, even if they were leading the main research activities. The number of Q&As and other Facebook events ranged from 26 (Mealtime Hostage) to 54 (BOBAB/UKBAPS group) depending on the needs of members.

## Results

### Demographics of PSG parents

722 parents filled in a pre-project survey. Based on this data, our typical member was

- 25–44 years old (> 90%)
- female (> 95%)
- white (~ 80%)
- a parent to 1 or 2 children (> 80%)
- based in the UK (~ 90%)

**Fig. 1:**
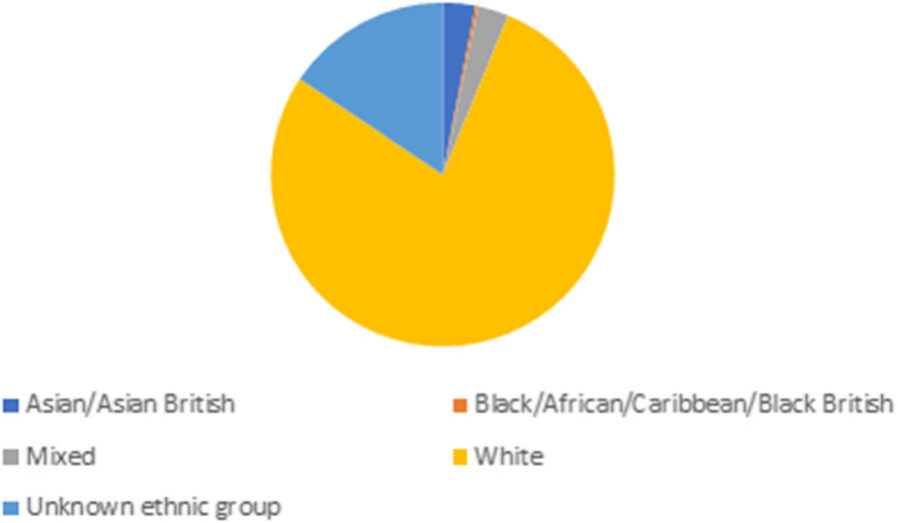
Ethnicity of participants (*n* = 715)

While this project was aimed at all parents, and despite a concerted effort on behalf of the project team ahead of the recruitment of Year 2 groups to look for groups which included fathers, the groups that our PSG groups were drawn from were predominantly female. It was difficult to analyse ethnic groups present in PSG, as many people did not wish to choose from the 2011 standardised ethnic categories provided by the Office of National Statistics. Many people chose to write in the free text box, and with a wide range of styles of entries, it was difficult to categorise accurately. For the 715 participants displayed in Fig. 1, we have used Wellcome’s reporting structure to define ethnic groups and only categorised individuals who positively identified themselves as being in these groups.

Typically, our members had a high level of science qualification compared with the general public - 63% of pre-project respondents and 72% of post-project respondents had a science qualification at undergraduate degree level or higher. While it is difficult to ascertain the exact percentage of science qualifications in the general public, in 2017, around 42% of the population aged 21 to 64 had achieved a higher education qualification of some type and for the last five years, around 40% of qualifications were in science areas. This gives an estimate of around 17% of the population with science qualification at undergraduate degree level or higher [49, 50]. Around 30% of our respondents worked as a scientist or had a science-related job and many more were typically highly engaged with science in their everyday lives. Fewer than 20% of respondents did not know anyone that worked as a scientist or were in a job that uses science.

### Overview of studies by PSG groups and collaborators

All of the groups identified a research question and conducted a study. The two breastfeeding groups, BOBAB PSG and UKBAPS PSG, worked as a combined group on two projects, making a total of eight projects. Table 2 outlines the research topic, the type of study, collaborators, the activities undertaken and the primary outputs that have resulted from the project at the time of writing. The emphasis of this paper is on the co-design process from conception to dissemination, though highlights of all projects and details of three projects are presented to illustrate the variation in study implementation and results. Detailed results of the full experiments are being disseminated by the researcher collaborators and group members, and a detailed analysis of the impact of the experience on PSG participants will be presented in another paper.

**Table 2:** Summary of the PSG groups, showing number of members and where they were recruited from, research topic, research method, names of collaborators, and summary of the research outputs

Name of group	Number of members	Recruited from	Research Topic	Research Method	Collaborator(s)	Research outputs
Science Aware Natural Parenting PSG (SANP PSG)	250	Science Aware Natural Parenting (Facebook group)	Effect of babywearing (carrying infant in a sling or wrap) on the temperature of a baby	Laboratory-based experiment using thermos-sensors with 9 mother/baby pairs	Dr Davide Filingeri, Loughborough University	Paper by PSG, Filingeri and two colleagues in review; PSG group member presentation to Midlands Baby Carrying Convention; Filingeri and PSG group member presentation at the Institute of Physics and Engineering in Medicine Physiological Measurements Special Interest Group
Dumfries and Galloway Bumps, Babies, Beyond PSG (DGBBB PSG)	80	Mummies United and other Facebook groups affiliated to DGBBB	Flexischooling	Collection of data through Freedom of Information requests, surveys and qualitative interviews	Dr Tara Jones, University of the West of Scotland	Paper based on PSG research in Other Education; group member and PSG Director, interview on Mornings with Kaye Adams, BBC Radio Scotland
Big Birthas PSG (BB PSG)	155	Followers of the Big Birthas blog by Amber Marshall, existing PSG groups, and by word of mouth	The effect of choice during pregnancy and labour for mothers with a high BMI	Qualitative study using email interviews	–	Report on thematic analysis on PSG website; paper in preparation; Primary Care & Public Health conference stall
Let Toys Be Toys PSG (LTBT PSG)	650	Open recruitment for LTBT supporters, advertised on social media and to PSG members	Gender stereotypes in children’s books	Intervention study including control group where parents read children books for five weeks	Dr Lauren Spinner, University of Kent	Results report and discussion published on PSG website
Breastfeeding and Health Care Experiences PSG (BF HCE PSG)	440	Year 1 PSG groups and then members of the wider breastfeeding community on Facebook	1) Breastfeeding and healthcare experiences 2) How personal experience of breastfeeding affects practice of health care professionals	1) Survey of mothers’ interactions with healthcare professionals analysed using corpus linguistics 2) Peer-to-peer qualitative interviews	1) Dr. Gavin Brookes, Lancaster University 2) Dr. Yan-Shing Chang, King’s College, London	Presented results at the Infant Feeding All-Party Parliamentary Group; Dr. Brookes has a book in preparation with a chapter based on project; presentations at the Association of Breastfeeding Mothers and the Institute of Health Visitors
Mealtime Hostage PSG (MH PSG)	327	Mealtime Hostage (Facebook community)	ARFID (Avoidant / Restrictive Food Intake DIsorder) and sensory sensitivity	Questionnaire followed by statistical analysis by collaborators	Dr Terry Dovey (Brunel University) Prof Jackie Blissett (Aston University)	Poster at British Feeding and Drinking Group Conference, and International Conference on Children’s Eating Behaviour; one paper published in European Psychiatry and another in progress
Breastfeeding Older Babies & Beyond PSG (BOBAB PSG)/ UK Breastfeeding and Parenting Support PSG (UKBAPS PSG)	1180 (total across both groups)	Breastfeeding Older Babies & Beyond (Facebook community) UK Breastfeeding and Parenting Support (Facebook community)	Constituents of breastmilk from mothers feeding older infants	Laboratory analysis of expressed milk via mass spectroscopy and microbiota analysis	Dr Natalie Shenker and Dr. Simon Cameron, Imperial College, London	Journal article in progress; presentations planned at the Association of Breastfeeding Mothers and the Institute of Health Visitors, additional presentations by collaborators at UNICEF Baby Friendly Initiative, and Metabolomics

### In-depth case studies

In the following section, we present a detailed description of the approach and implementation of three of the projects to illustrate the range of topics covered through our radical co-creation project, how participants and collaborators interacted, and some preliminary findings of the projects to demonstrate the scientific value of co-producing research.

### Project 1: Breastmilk composition

Two of the ‘mother’ groups used to find members of the PSG Facebook groups were breastfeeding support groups. ‘Breastfeeding Older Babies and Beyond’ (BOBAB) is for mothers feeding children older than one year. It is a UK-based group, although there are a significant minority of non-UK members. The UK has one of the lowest breastfeeding rates in the world and despite WHO recommendations to breastfeed babies for 2 years, only 0.5% UK children are getting any breastmilk at 12 months old [51]. The mothers in this group are therefore atypical, perhaps more committed to or more informed about breastfeeding than average.

The term ‘extended breastfeeding’ is used in many contexts to refer to feeding past one year, but the group tend to prefer ‘natural term breastfeeding’ as a less loaded term and one that is being increasingly adopted [52]. Many conversations in the group reflected the experiences of many mothers that breastfeeders of older babies and children can face negative judgements from family members, strangers when feeding in public, and from healthcare and childcare professionals [53]. These pressures from “social surveillance” mean many women, especially in Western societies, may stop breastfeeding children even when they would prefer to continue [54]. Members in the BOBAB group therefore particularly appreciated a ‘safe space’ where longer term breastfeeding is treated as normal.

A significant minority of the BOBAB members are not currently breastfeeding, but work as lactation consultants, or similar roles supporting breastfeeding mothers. For many, breastfeeding is part of their identity as mothers. Some members are mixed feeding or have formula fed previous children.

The second Facebook group involved in PSG is the UK Breastfeeding and Parenting Support (UKBAPS), a general breastfeeding support group with no restrictions on the age of child, so people tend to join when their child is a young baby. There are no demographic details available, but the impression of PSG staff (and of the admins of both groups) is that the members of UKBAPS are, on average, younger and less educated than BOBAB. There are posts in the group from people feeding children over one year, but there are also many posts about newborns and babies a few months old.

This group also had numerous posts from members talking about pressure to stop breastfeeding or negative judgement from others. One common occurrence in both groups was that a member visits their GP about a medical issue unrelated to breastfeeding. They are prescribed medication and they ask if it is breastfeeding-safe resulting in the GP responding, ‘But your baby is X age, don’t you know that there is no benefit to breastfeeding past 6 months/1 year/18 months?’

Discussion threads prompted by these incidents raised the question, “Why are they saying this when there isn’t any research on what’s in breastmilk at this age?” and then followed by, “WHY isn’t there any research on what’s in breastmilk past 18 months?” At the time of our project, there was no published research on the composition of breastmilk past 18 months postpartum, and very little past 12 months [55].

In the early months of the project, the groups had joint online Q&As with various breastfeeding and breastmilk experts. One of these was Dr. Natalie Shenker, Imperial College London. She told the group that she was hoping to conduct research on the composition of breastmilk for older babies and the idea for a collaboration was born.

Both groups came up with dozens of questions they thought would be interesting to investigate. After discussion and voting, both groups chose the question “What is the composition of breastmilk for children over 2?” by a considerable margin. The groups therefore decided to pool their resources and work together on this experiment, collaborating with Natalie. She made contact with her colleague Dr. Simon Cameron from Imperial who was developing a novel mass spectrometry technique for profiling of microbial communities and was looking for samples to test [56].

In collaboration with Natalie and Simon, the group developed a protocol for a cross-sectional study using rapid ionisation mass spectrometry (REIMS) to investigate the composition of breastmilk from mothers feeding infants in six age categories (0–6 months, 6–12 months, 12–24 months, 24–36 months, 36–48 months and 48+ months). Once the protocol was agreed, Natalie was able to submit it as an amendment to ethics approval for an existing large scale study. Once ethics approval was in place, a suitable date was identified and the Facebook groups, supported by the PSG staff, began organising the recruitment and logistics. Group members were very keen to volunteer, but many of them couldn’t come to London to donate breastmilk for various reasons. To increase numbers, mothers were recruited from elsewhere, including ‘mother groups’, local breastfeeding support groups, and personal networks of members.

130 mothers who were breastfeeding children across the age ranges came to Charing Cross Hospital on 21st February 2018 from as far away as Scotland with 117 able to express milk on the day. The participants congregated in the students’ union bar for the day where mothers (and accompanying children) could gather before and after their slot to express. Group members not only donated their breastmilk, but they helped with logistics on the day - bringing toys for babies to play with, distributing questionnaires, entertaining children, delivering samples to Simon’s team, and so on.

Samples were analysed using REIMS to identify components, and characterise the microbiome, and fat content. Preliminary results suggest that of the approximately 6900 different components in the breastmilk samples, around 150 of them seemed to vary systematically by the age of the nursling. Analysis is currently ongoing by the collaborators and results written up for publication.

### Project 2: breastfeeding and healthcare experiences

Many discussions in the two breastfeeding groups in year one centred around why UK breastfeeding rates are so low. One theme that came up repeatedly was people’s interactions with healthcare professionals (HCPs). Members put forward various theories on how HCPs impact on breastfeeding journeys, and people gave anecdotes, either personal, or from their own experience as breastfeeding peer supporters, midwives, GPs or other healthcare providers. The idea emerged from several discussions that members would like to form a new project looking at people’s experiences with healthcare providers around breastfeeding.

In year two, PSG project staff created a new group and invited members from the two breastfeeding groups, from any other PSG group who wanted to join and from the breastfeeding mother groups. We also encouraged members to invite friends they thought would be interested, and share with other breastfeeding support groups on Facebook. The group ended up with 440 members, all of whom are or were breastfeeding mothers. Roughly 50% of them were also breastfeeding peer supporters or healthcare professionals, including nurses, midwives, GPs, paediatricians and health visitors. The HCPs were often concerned about the level of training about breastfeeding in their profession. Many expressed the view that they hadn’t known anything about breastfeeding until they were doing it themselves, and then they realised how misguided much of their previous advice to patients had been.

After many discussions in the group and online Q&As with relevant researchers, the groups decided they wanted to do two studies:

1. Looking at mother’s experiences and the effect of interactions with HCPs on their breastfeeding journeys.
2. Looking at how HCPs’ experiences of becoming mothers and breastfeeding themselves changed the advice and support they gave to patients

#### Study 1

Working with Dr. Gavin Brookes, University of Lancaster, and with advice from various other researchers through Q&A sessions, the group designed a survey to gather stories of the infant feeding journeys of mothers who breastfed or who wanted to breastfeed, and their interactions with HCPs. They shared this survey invitation with the mother groups, and other breastfeeding support groups, and with their personal networks. Mothers clearly wanted to tell their stories. The post inviting people to fill in the survey was titled, “We want your stories on infant feeding and healthcare!” And the first reply said, “I feel I have been waiting for this day for six years.”

In total 741 infant feeding narratives comprising 257,319 words were collected, along with data including age of mother and child, ethnicity, type of HCP in the interaction and details of what happened after the encounter. The text was then analysed by a team of group members using corpus linguistics techniques and free AntConc software [57]. A preliminary summary of their findings is shown in Table 3.

**Table 3:** Preliminary analysis of interactions between breastfeeding mothers and healthcare professionals

*Issues*	*What worked / what mothers wanted*
● The early days are crucial. ● “Throw away” comments can ‘make or break’ breastfeeding for many mums. ● Lack of time for staff is a major factor. ● Tongue tie seems to be commonly missed or down-played. ● Problematic latches and the effective transfer of milk were a significant issue but often the focus was on treating weight loss, thrush, mastitis or nipple damage rather than the underlying problem.	● Listening to the mother is the most important thing. ● The success stories normally happened when one person listened to the mother about the whole situation and worked through everything step by step. HCPs need to be ready to listen to what mothers want/need before offering any advice. ● HCPs working with mothers should be prepared to admit they don’t always know the answer and be willing to look in to it

Group members found it emotionally affecting to analyse these narratives, but ultimately empowering to be doing this research. Some of the comments from those doing the analysis included:

“I cried reading many of the stories and then I felt angry on behalf of all the failed mothers!”

“I am so glad this [research] was happening and I could do something constructive.”

“I really hope that people will listen to us about this. Reading the stories was incredibly powerful for me, but we’ve got to try to find a way to get that across to the people that can make changes.”

Gavin Brookes is currently writing a book on analysing health language data which will contain a chapter based on the project.

#### Study 2

The second study by this group investigated the effect of the health care professionals’ own breastfeeding experiences on the support that they offered breastfeeding mothers. It.was devised and run with support from Dr. Yan-Shing Chang of Kings College London and workshops were facilitated by Dr. Petra Boynton, independent researcher and author of The Research Companion. The group recruited from within their membership HCPs who were involved in breastfeeding support at work. In pairs, they interviewed each other and a team of PSG volunteers analysed the interviews using thematic analysis. Eight HCP interviews were conducted, including a consultant paediatrician, a GP and several nurses.

##### Preliminary Results.

The preliminary results of the analysis based on experiences of health care professionals before and after breastfeeding are summarised in Table 4.

**Table 4:** Preliminary results of experiences of health care professionals before and after breastfeeding

*Before breastfeeding experience*	*After breastfeeding experience*
● Little formal training on breastfeeding ● Lack of knowledge of breastfeeding norms ● Familiar with myths on when to wean e.g. when the child gets teeth, or can talk, or is just “too old” ● Feelings of discomfort around breastfeeding mothers	● Positive experiences of breastfeeding to sleep conflicted with some mainstream baby sleep advice ● Keeping mothering practices (e.g. natural term breastfeeding) secret from colleagues for fear of disapproval: ● HCPs’ work environment not breastfeeding friendly for mothers who return to work while breastfeeding ● Important informal knowledge gained from own experience, support groups, online communities and observing other mothers ● Participants became ‘breastfeeding champions’ ○ Participants used as informal breastfeeding experts by colleagues ○ Used knowledge of normal breastfeeding and sleep behaviour to support mothers ○ Able to recognise and confidently challenge breastfeeding unfriendly practice

Presentations on the results of both studies in this project were made at the Association of Breastfeeding Mothers, the Institute of Health Visitors and the Infant Feeding All-Party Parliamentary Group.

### Project 3: mealtime hostage

In the second year of the project, existing parenting groups were invited to apply to take part. One group was Mealtime Hostage, a primarily US-based group with almost 13,000 members, who are parents of children who are selective eaters. Some are fairly typically developing children who are going through a food neophobic stage, but some have children who are far more severely restricted in their diets. Many have been diagnosed with avoidant/restrictive food intake disorder (ARFID) and some are so restricted in their eating that they have ended up on feeding tubes or have only one or two ‘safe foods’ (i.e. foods they will reliably eat).

Several experts participated in online Q&As with the group and two of these, Dr. Terry Dovey (Brunel University) and Prof Jackie Blissett (Aston University), offered to collaborate with the group on their research. Terry took the lead with the groups, and oversaw the research including gaining ethics approval through his institution while Jackie provided advice and feedback.

The overriding goal of the group is to find effective interventions that will help children with ARFID and similar conditions, and to establish early and accurate identification of these children. However, their discussions with experts quickly showed that research into these conditions was still in its infancy and that intervention research could not be carried out until the conditions themselves were better understood.

Discussions both within the group and with Terry and Jackie brought up that many members’ selectively eating children had strongly disliked certain textures and could be more anxious than their peers about new places and situations. The group wondered if these were common features and if they might be underlying causes for the eating behaviours.

Working with Terry and Jackie, the group decided to investigate the emotional behaviour (e.g. anxiety) and sensory sensitivity of children with a range of eating issues. The researchers helped the group to select validated measures that could be combined to create a questionnaire including the Behavioural Paediatrics Feeding Assessment Scale and the Child Eating Behaviour Questionnaire. After gaining ethics permissions from Brunel University, the group publicised the survey, primarily through the main Mealtime Hostage group, and also other groups for parents of selectively eating children, as well as on their personal newsfeeds and though their networks.

The questionnaire was viewed by 1238 people resulting in 551 usable submissions. Terry took the lead on analysing the data, but to keep the group as involved as possible, he recorded a video of him exploring the data on Qualtrics for the first time and explaining what he was looking for and what he could do with the data. He was particularly excited that the respondents covered a significant number of children with an ARFID diagnosis and other diagnoses like Autism Spectrum Disorder, as well as typically developing children, and those with parent-reported eating issues but no diagnosis. His comments included “This is an awesome data set. I have never seen in my career to date a data set like this. You’ve managed to do something that we [academic researchers] haven’t thus far, which is get a good sample of people with different diagnoses, and controls, answering the same questions. This is massive....brilliant ...... I don’t know anyone in science [on this topic] who has this.”

He discussed the need for data cleaning, e.g. ‘age of child’ had been entered in a free text box, so would need converting data such as ‘3.5 years’, ‘3 years 6 months’, ‘3yrs6m’ into categorical data. The group immediately offered to help with this and Terry shared individual columns of data on Google Docs where group members coordinated cleaning it. By the next morning all the problem columns had been corrected and analysis could start.

Preliminary findings include that children with an ARFID diagnosis appeared to have more visual and tactile sensitivities than other groups and that children with parent-reported feeding issues appeared to be on a continuum of scores across the scale. Score profiles showed similarities with the ARFID diagnosis with the highest scoring children being the ones that ultimately might get a diagnosis. The first paper based on this collaboration has just been published in European Psychiatry, naming the group as a co-author [58]. Prof Blissett and Dr. Dovey presented posters at the British Feeding and Drinking Group Conference and the International Conference on Children’s Eating Behaviour and several more papers based on the research are in preparation.

### Outcomes for PSG participants

A survey pre-project (722 responses) and post-project (276 responses), with 85 participants filling in both, asked about awareness of and confidence in science-related areas. All of the respondents in both surveys said they had someone to talk to about science and over 90% felt confident in their understanding of science stories in the news. Over 70% of respondents mentioned evidence or research as the primary reason they would trust a source.

However, other positive benefits did occur which were captured by qualitative questions in the post-project survey. When asked “What, if anything, did you feel you got out of being involved in PSG?”, there were 172 free text responses mentioning specific gains. Of these, the most common (28 times) was a ‘Sense of community’ which was about feeling a part of a group of people with the same goals and challenges, for example “a feeling of being part of a community of people working for a similar cause” and “It’s been really lovely to be part of a very similarly minded group of parents who all understand your home situation and can make allowances for babies and toddlers, but still produce some amazing results”. The second most mentioned categories were ‘Opportunity to add to knowledge/change practice’ and ‘Knowledge gains’ with 23 mentions each, for example “Got to be on the cusp of new science! That was cool” and “Proud to be part of something thats results could lead to fairer treatment of bigger mums”. The fourth most common was ‘Increased confidence’ (20 times) either about confidence in their own parenting choices or about being able to understand research evidence and to explain it to others who might question their decisions. One member responded with “[the project] has increased my confidence in speaking out about breastfeeding views & issues. It’s so great to be able to respond to negative/uninformed comments with “Well I was part of an experiment & we found this result...“ & have an air of confidence and authority, rather than just mumbling ‘Well I’ve read an article on Facebook...’!”. While most responses were very positive, there were a few negative ones. Five people felt that they couldn’t contribute because they didn’t have time, four said they just didn’t get much out of the projects and two said they didn’t feel included because their group was “clique-y”.

Members of PSG projects often mentioned appreciating the chance to have intelligent conversations with other adults, especially other mums who were going through the same experiences, and to work together as a community to achieve something, particularly in the context of mothers with young children feeling isolated, starved of adult interaction and as if they had suffered a loss of their identity and confidence. One respondent said a strength of the project was “Parents! Parents asking the questions we want the answers to but no-one thought to ask! Recognising that while we may be Mums with baby-brain and dubious stains on our clothing, that we’re still capable, intelligent beings and we have a lot to contribute!”. A small but telling set of responses were the seven parents who said that PSG had influenced their career direction, ranging from embarking on further study in science to returning to or re-focussing their science career prompted by their experience.

The ‘experts’ involved in the project were also impacted by their participation, as evidenced through surveys sent after the Q&A sessions. One scientist noted “Being part of PSG informs the way I want to conduct science, but also the way I engage with information outside of my own subject area”. Many mentioned great satisfaction in finding and addressing research gaps that were important to parents. The projects gave some scientists new ideas and different perspectives on their work: “It was great to be able to have a multi-way conversation and get other people’s perspectives on my work” and “[Some of the] questions that were being asked potentially would make good research questions, as the field is very under-researched”. Other collaborators said they were inspired to do more public engagement, for example “The importance of public engagement has been emphasised to me and I am trying to incorporate this further into my work day”, and appreciated hearing from PSG members that their research was useful and interesting.

## Discussion

### Methods of engagement

There are many approaches to engaging interested parties in research, ranging from minimal input via electronically mediated consultations or single community meetings to full participant control of projects. As described in Arnstein’s Ladder of Participation [59], the goal of PSG was to push the involvement of parents up the ladder, depending on the project, beyond *consultation* to *partnership* where power and responsibility is shared between parents and experts, such as in the breastmilk composition study, and when possible to full *participant control* where parents handle the entire project from question exploration to data gathering to analysis with support and facilitation, as in the Big Birthas study.

How radical was PSG? By developing recruitment methods using Facebook to identify potential groups and to facilitate engagement, we were able to achieve the goals of fully engaging the target public of parents of young children in the co-design of scientific studies while not creating a burden on time or restricting participants due to disability, financial status or living in a particular location. Participants were able to choose how much or how little to be involved, but all still had the opportunity to contribute. All eight of the PSG groups were able to engage in meaningful discussions with experts and lead a study on the questions of interest to parents. We set out to co-produce the research and remove any hierarchy between ‘expert’ and parents, with PSG members deciding what topics to pursue and therefore what type of researchers we needed to engage to help us answer our questions. This meant that the researchers had not necessarily co-produced research with members of the public before, and we had a few instances of differing expectations of how the partnership should work, and on occasion this led to frustrations from PSG members when they felt they were not as in control as they would have liked.

### Effects on participants

At the point of writing, the PSG projects have led to at least eight papers in press or in preparation and seven conference presentations, and presentations to Members of Parliament, with more outputs to come (see Table 2), and several experts commented that engaging in the project had opened up new areas of research for them that they would not have otherwise explored. While the scientific outputs are impressive, PSG also had the goal of changing participants relationship to science and increasing *science capital*, providing support and scaffolding along the way to ensure that the participants gained confidence in understanding research and evidence. Science capital is defined here as level of science-related qualifications, understanding and knowledge of the scientific process, interest and science-related contacts [60]. Our pre-project questionnaire showed high levels of scientific qualifications and awareness of science in the groups, and so it is unsurprising that we did not see evidence of significant changes reflected in the quantitative questions before and after the projects in terms of understanding of and engagement with science. The over-representation of people with high levels of education mirrors citizen science projects more widely [61], as does the lack of demonstrable change related to general increase in understanding or engagement with science. For example, Jordan et al. [62] note that although citizen science projects are often touted as a way of increasing general scientific knowledge and literacy, observed effects tend to be restricted to specific content knowledge.

### Health topics in co-designed experiments on parenting

Most of the projects had a health related theme and many of the discussions started with expressions of dissatisfaction with the treatment of parents by health professionals or the feelings of being disempowered and marginalised by pregnancy and motherhood as well as the lack of evidence for many of their questions and concerns. The Parenting Science Gang approach meant that we could take these frustrations and channel them into actions to have the voices of our participants heard. For example, the projects on breastfeeding experiences involved health professionals who had a ‘foot in both camps’, allowing a more nuanced and constructive engagement than many of the parents had experienced before in their health care encounters. Several of the HCPs became advocates for breastfeeding mothers in their workplaces, supporting them in their wishes and helping to make the healthcare settings more breastfeeding friendly. The results of this project around mothers’ experiences with HCPs and the experiences of HCPs themselves were presented on 16 July 2019 during a lively and very positive session, attended by PSG members and their children, at the Infant Feeding All-Party Parliamentary Group who are particularly interested in increasing breastfeeding rates and support for breastfeeding mothers.

### Caveats and limitations

The PSG was a learning curve for everyone involved. It was necessary to find the best use of Facebook and associated tools in a manner that works for the researchers and participants. Facilitators need to be skilled and confident so that the experts do not dominate, to manage the expectations of experts and parents about what can be achieved, and to help manage the process for agreeing which question out of hundreds submitted would be chosen while not alienating those whose topic was not picked.

This radically user-led design meant that the PSG staff had to live with a high degree of uncertainty. We did not know what topics the groups would choose to research, what kind of experiment they would want to do, what the costs would be or how long it would take, all of which made advance planning almost impossible. In most cases, we could not book experts for Q&A sessions months in advance, because we had no idea what they would be talking about. The PSG approach made gaining ethical approval in a timely fashion a major challenge with most ethics panels expecting applications weeks or even months before a study would begin. Practical work such as the considerable logistics for the breastmilk experiment - getting 130 nursing mothers plus children to the same hospital in London, on the same day, with all the equipment and paperwork needed - couldn’t be started until the groups had agreed the protocol. To deliver the ambitious goal of co-design of studies from beginning to end necessitates a willingness to live with uncertainty on the part of the research staff and the funders in order to truly hand over power to the participants.

The project was unsuccessful in recruiting many parents from social groups such as ethnic minority or lower socioeconomic members or those who were not already engaged with science in one way or another. We attempted to engage with under-represented groups in year 2, by asking PSG members for recommendations of Facebook or offline groups we could approach, but this was not successful in terms of establishing new PSG groups. One of the challenges we encountered when trying to engage with minority groups was that large Facebook groups focused on them do not exist, at least in the UK, and this was our main recruitment method. The small grassroots community organisations that exist to support, for example, black and minority ethnic parents, simply don’t have the capacity to engage with projects such as ours. Building deep and trusting relationships with people takes significant time and resource, particularly for community-level organisations who may be skeptical of new people. Future work will include efforts to engage with more diverse groups, building in funding to support face-to-face visits with community and support organisations to explore the best platform to engage in discussions and projects with them more effectively. Grants need to include funding to support community organisations to engage with projects.

## Conclusions

The importance of the Parenting Science Gang approach presented in this article is to show the radical nature of truly allowing the ‘public(s)’ to determine the study from beginning to end and to participate in all aspects of the research. The approach has been shown to be possible through the multiple projects of PSG, but requires effort and organisation by the facilitators and a great deal of flexibility on the part of the experts involved. The distributed and voluntary nature of Facebook allowed more parents, the target public, to be involved as much or as little as they wanted or could be, empowering them by handing them the power to set the agenda rather than offering the chance to have selected input into a study where the topic and the research leads have already been decided, as in more traditional PPI projects. This resulted in a transformative relationship for many of the parents and experts, giving those scientists who were willing to be in a supporting role for the decision-making the opportunity to work on topics that they may not have considered before and to gather robust data with the assistance of the participants.

The Parenting Science Gang has achieved its goals of producing successful and academically productive radically co-designed projects, but as or more important from our point of view is the positive effects it has had on many of the participants, parents and experts. We end with a quote from one of the participants that reflects the potentially life-altering impact that this approach can have:

“I found new areas of interest and reawoke old ones. I’ve made friends and had my faith in myself and abilities restored. I can still use my brain and read academic texts (and science ones at that!) and have intelligent, grown up conversations! I’ve been waffling about returning to work and what I could do, I suddenly have more ideas and enthusiasm thanks to working with PSG and meeting the others involved.”

## Supplementary information

**Additional file 1.** GRIPP2 Long Form PSG. GRIPP2 Long Form Table of Criteria for Parenting Science Gang Project.

## Data Availability

The pre- and post-survey datasets presented in the published article are available from the corresponding author on reasonable request. Datasets of individual projects are the property of the lead experimenters.

## Abbreviations

ARFID: Avoidant/Restrictive Food Intake Disorder
BB: Big Birthas
BFHCE: Breastfeeding Health Care Experiences
BOBAB: Breastfeeding Older Babies and Beyond
DGBBB: Dumfries and Galloway Bumps Babies and Beyond
GP: General (medical) practitioner
HCPs: Healthcare professionals
IAP2: International Association for Public Participation
LTBT: Let Toys Be Toys
MH: Mealtime Hostage
PPI: Patient and Public Involvement in Research
PSG: Parenting Science Gang
Q&As: Question and answer session
REIMS: Rapid ionisation mass spectrometry
SANP: Science-Aware Natural Parenting
UKBAPS: UK Breastfeeding and Parenting Support
